# Vacancies for Midwives

**Published:** 1916-04-15

**Authors:** Meredith Young

**Affiliations:** County Medical Officer. 43, Foregate Street, Chester


*To the Editor of* The Hospital.

### Vacancies for Midwives.

*To the Editor of* The Hospital.

Sir,—It may interest some of your readers to know that there is a distinct shortage of midwives in certain parts of this county, notably in Nantwich Urban District (population 7,900, three midwives, two of whom are very old and will shortly be giving up practice), Tarporley Urban District (population 2,600, no midwife), portions of the Chester Rural District (Barrow, Ince, Hapsford, Dunham Hill, etc.), Congleton Rural District (Mow Cop and Mount Pleasant, population about 1,500), Macclesfield Rural District (Rainow, population 1,200; Adlington, population 700), and Wirral Rural District (Thornton Hough, population 650).

If any of your readers should care to write me I will gladly supply any particulars in my power.—Yours faithfully,

Meredith Young, County Medical Officer.

43, Foregate Street, Chester, April 7, 1916.

